# Cardiac MRI after Sudden Cardiac Arrest in a Young Woman Prompts Diagnosis of Familial Dilated Cardiomyopathy

**DOI:** 10.5334/jbsr.3064

**Published:** 2023-03-30

**Authors:** Nico Hustings, Valerie Van Ballaer, Leticia Barrios

**Affiliations:** 1 UZ Leuven, BE

**Keywords:** Cardiac MRI, echocardiography, familial dilated cardiomyopathy, ventricular arrhythmia, sudden cardiac death, TTN gene mutation, late gadolinium enhancement, non-ischemic LGE, myocardial fibrosis

## Abstract

**Teaching Point:** Familial dilated cardiomyopathy (DCM) predisposes to malignant ventricular arrhythmias and sudden cardiac death, and magnetic resonance imaging (MRI) has important diagnostic value in demonstrating non-ischemic patterns of late gadolinium enhancement (LGE).

## Case History

A 26-year-old woman was resuscitated following sudden cardiac arrest caused by ventricular fibrillation. The personal medical history was unremarkable. However, the family history revealed a twin brother with idiopathic dilated cardiomyopathy (DCM).

**Figure 1:**
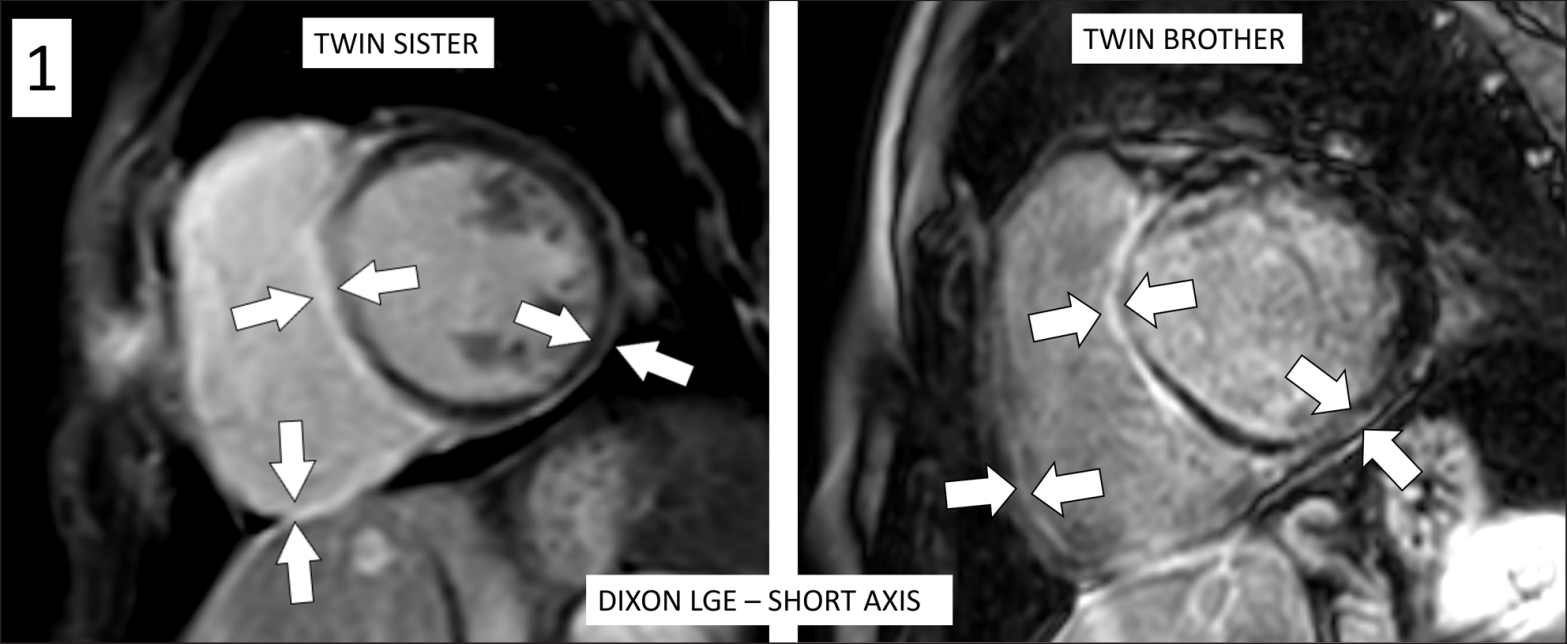


**Figure 2:**
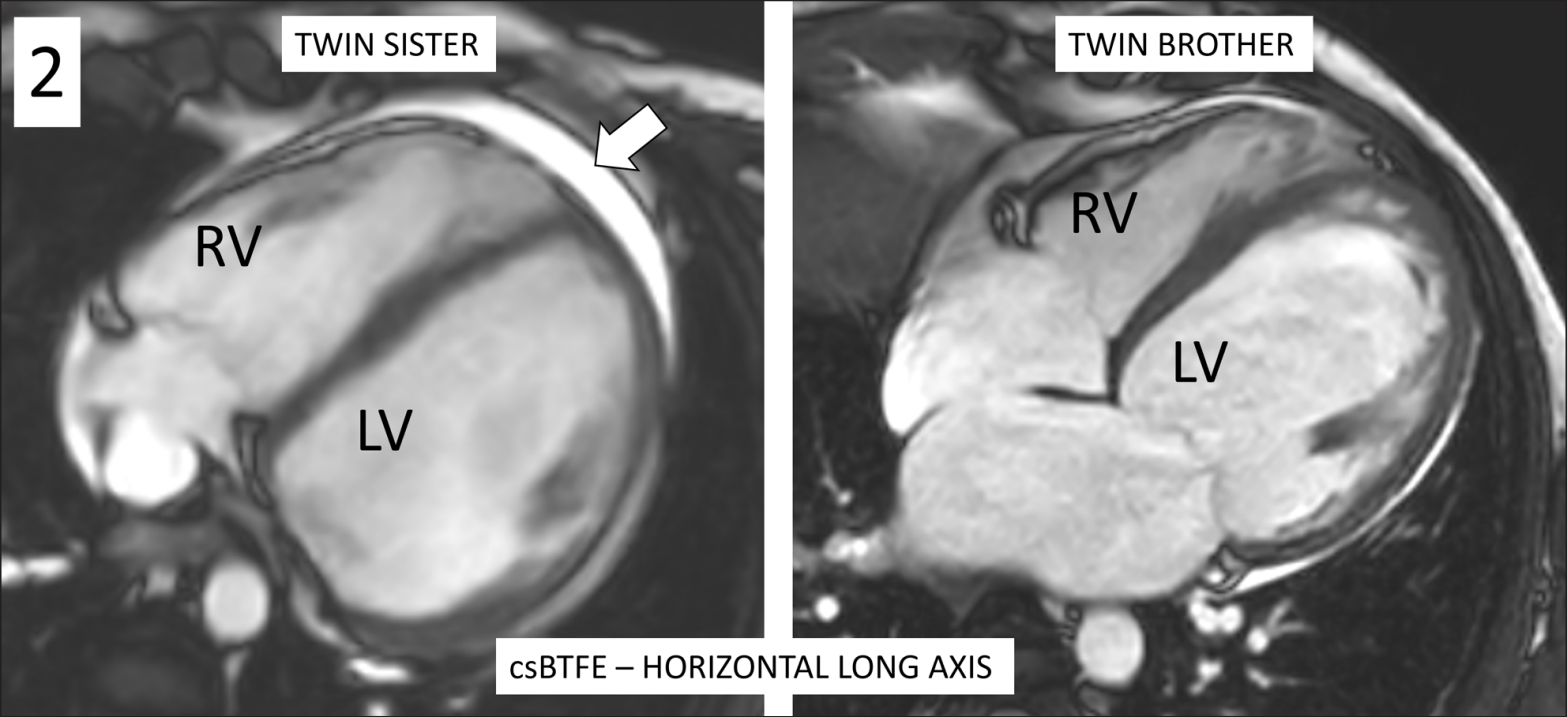


**Figure 3:**
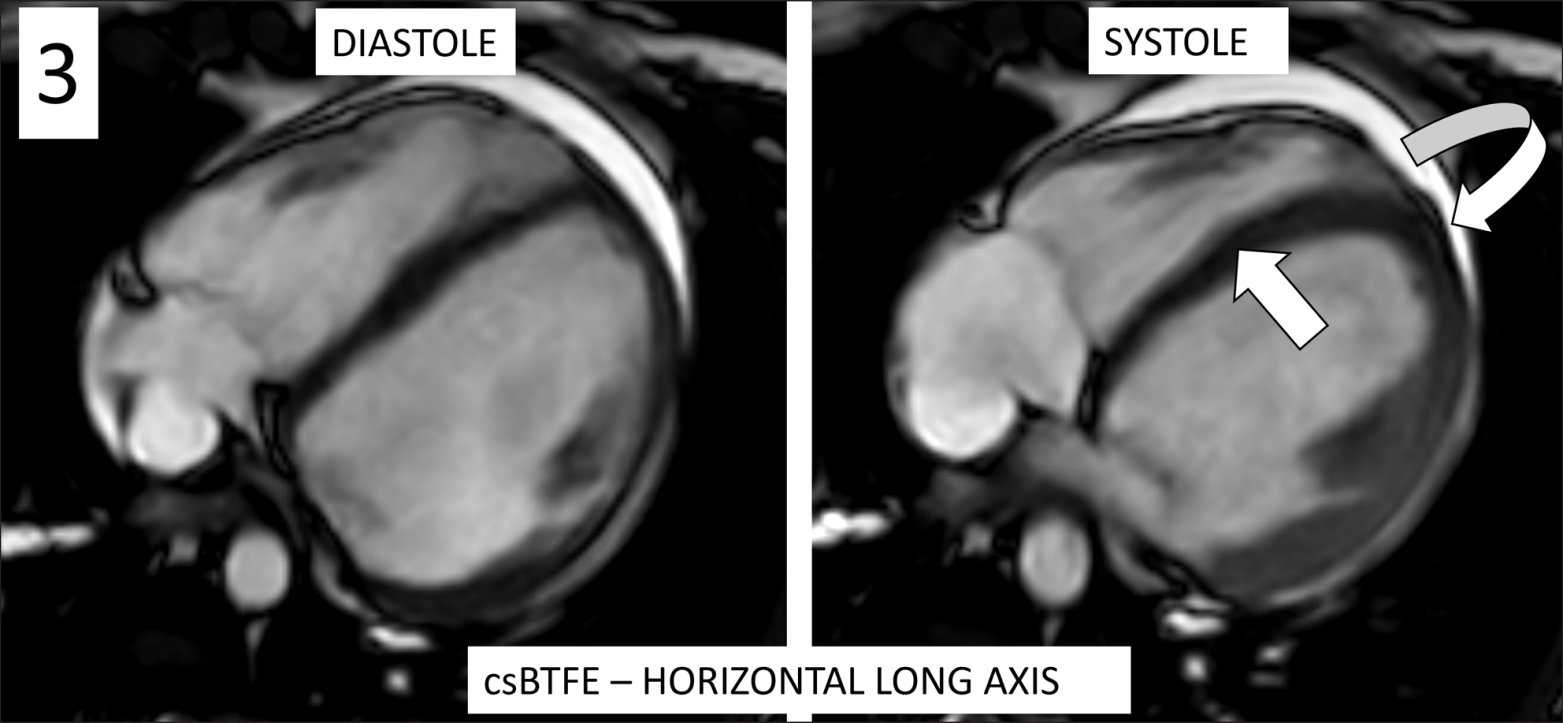


Echocardiography revealed mildly dilated ventricles, moderate right and severe left ventricular dysfunction, and asynchronously contracting left ventricle with apical rocking and septal flash. Cardiac MRI depicted biventricular dysfunction, particularly left-sided with ejection fraction ±30%. Figure 1 demonstrates extensive biventricular myocardial late gadolinium enhancement (LGE) in a non-ischemic subepicardial ring-like pattern (white arrows) on the magnetic resonance imaging (MRI) of the patient, with similar LGE pattern on the MRI of the twin brother. Figure 2 shows mild dilatation of the ventricles, even more pronounced in the twin brother. A small pericardial effusion (arrow) is seen in the sister. Video 1 demonstrates the apical rocking and septal flash on the MRI of the twin sister, also illustrated by Figure 3: systolic apical rocking (curved arrow) and septal motion away from the left ventricle (straight arrow).Consecutively performed genetic testing confirmed that the patient and her twin brother were carriers of truncating mutations in the titin (TTN) gene, leading to familial DCM. Interestingly, this identical gene mutation led to similar myocardial LGE patterns in individuals. The patient received cardiac resynchronization therapy (biventricular pacemaker) with defibrillator (CRT-D) for heart failure and secondary prevention of ventricular fibrillation. She also received medical treatment consisting of betablockers, angiotensin receptor/neprilysin inhibitor, sodium-glucose-cotransporter-2 inhibitor, and a potassium-sparing diuretic.

**Video 1:** Apical rocking and septal flash.

## Comments

Estimated prevalence of DCM is 1:250 of which 30–50% consists of familial DCM [1]. Multiple genes are associated with familial DCM and 20–25% of cases are associated with truncating-TTN-mutations. The clinical presentation of DCM varies from asymptomatic to heart failure or sudden cardiac death. DCM is characterized by left ventricular systolic dysfunction (left ventricle ejection fraction <45%) and left ventricular dilatation, excluding any known cause of myocardial disease. Echocardiography is vital in the diagnosis, as well as follow-up and family screening of familial DCM by evaluation of ventricular volumes and function. MRI has added value in tissue characterization via LGE. The structural changes in DCM are associated with myocardial scar formation, presenting as LGE with a non-ischemic, that is, subepicardial/midwall pattern on MRI. The extent of LGE is predictive for malignant ventricular arrhythmias or left ventricle reverse remodeling [1].

According to the 2022 ESC guidelines for ventricular arrhythmias, genetic testing is recommended in patients with DCM and atrioventricular conduction delay at ≤50 years of age, or those who have a family history of DCM or sudden cardiac death in the first-degree relative (at age ≤50 years).
